# Around the Hospitals

**Published:** 1894-10-27

**Authors:** 


### AROUND THE HOSPITALS.

[Notice to the Managers of Institutions.—Special space is now reserved for the insertion of notices of hospital meetings and festivals. The Editor requests that all notices may reach The Hospital Office, 428, Strand, at least one week before the date of each meeting.]

**The Johannesburg Hospital, South Africa.**—Hospital treatment and arrangements in South Africa are becoming of general interest in England. The Johannesburg Hospital aspires to a position side by side with hospitals in the mother country, and its report finds its way through the same channels into English hands. The Johannesburg Hospital has had to fight with many difficulties, but these have been bravely met, and its resources have been generously supplemented by the Government. The Hospital Saturday movement has penetrated even to South Africa, and a collection made last year secured £1,899 for the hospital. Since the establishment of the institution continuous enlargement has been necessary, calling for additional supply of funds. In 1839 an east wing was added, with covered way between the convalescent room and the kitchen block, and new gates were erected. These were followed by the commencement of laundry quarters, nurses' quarters, drainage system, and renovation of main entrance road. The resident surgeon reports that further accommodation is necessary, both in the in and out patients' departments. During 1893 nearly half the patients treated paid full fees, the rest being received free or for part payment. The number of in-patients received amounted to 2,183, and the out-patients to 843. Of the in-patients 1,456 were whites and 727 blacks. A large number of the patients come from the mines. It is noticeable that the percentage of deaths from all causes was much larger amongst the black than the white patients. The income amounted to £21,181; the expenditure to £13,320. The cost per patient is given at £92 11s. The hospital is supported by a loan from Government, native registration fees, paying patients, and voluntary contributions.

**The Ingham Infirmary, South Shields.**—Amongst the various items which have added increased amounts to the funds of the infirmary during the past year subscriptions from the working classes rank high in importance. Indeed the income from this source amounted to more than one-third of the total revenue. Working men are represented in the management, and with this added responsibility interest in the institution is not likely to diminish amongst them. Instances of public interest are otherwise visible, also church and sunday-schools contributing increased amounts during the same period. Several legacies also show a larger total than they have done for some time past. Various changes have occurred in the medical department and management, both by death and retirement. The number of patients treated in the past year amounted to—in-patients, 265; out-patients and casuals, 7,961. The income amounted to £1,950 and the expenditure to £1,793. Whilst the funds are in so satisfactory a condition activity and care is manifested in the work and management of the infirmary. The largest number of in-patients yet received were treated; the expenditure, on the other hand, shows a decrease, being, in fact, the smallest recorded during the last five years.

**The Clayton Hospital, Wakefield.**—The report presented by the hospital this year is generally satisfactory. No very important events have marked the period saving the unusually large share of legacies which have been received by the institution. These legacies consisted of a sum of £20,000 through the will of Mr. Samuel F. Harrison, of £90 from the late Charles Martin, of £250 from the late George Alder, and an intimated legacy of £1,000 from the late Sellers Higgins. The funds otherwise have not been unusually well replenished. Subscriptions and donations show a falling off, but the congregational collections were larger. The

Hospital Saturday Fund collection, in spite of the unsettled state of the coal trade, was larger than was anticipated. The hospital is about to possess a portion of the vacant land opposite it, it being deemed advisable to secure this against other purchasers. It is gratifying to note that whilst the number of inmates has been larger during the past year, the care exercised by the matron has caused a reduction in household expenditure. The in-patients numbered 571 and the out-patients 3,742. Very little activity is visible in the convalescent branch. The Mackie Convalescent Fund provides an income of over £22 yearly. There are accumulated sums amounting to upwards of £80, yet the expenditure for 1893 was under £5. Three patients only received the benefit. The hospital patients of Wakefield must be unusually fortunate, both in their circumstances and surroundings, to need so little after assistance. How many other less fortunately situated hospitals both in the metropolis and elsewhere would thankfully avail themselves of these funds now lying dormant, to assist the recovery of many poor patients. During the year the income received amounted to £2,820 (less legacies); the expenditure was £3,002.

